# Overcoming the Blood–Brain Barrier: Advanced Strategies in Targeted Drug Delivery for Neurodegenerative Diseases

**DOI:** 10.3390/pharmaceutics17081041

**Published:** 2025-08-11

**Authors:** Han-Mo Yang

**Affiliations:** Division of Cardiology, Department of Internal Medicine, Seoul National University Hospital, 101 Daehak-ro, Chongno-gu, Seoul 03080, Republic of Korea; hanname@gmail.com; Tel.: +82-2-2072-4184

**Keywords:** blood–brain barrier, nanomedicine, receptor-mediated transcytosis, focused ultrasound, lipid nanoparticles, exosomes, CRISPR, neuroinflammation

## Abstract

The increasing global health crisis of neurodegenerative diseases such as Alzheimer’s, Parkinson’s, amyotrophic lateral sclerosis, and Huntington’s disease is worsening because of a rapidly increasing aging population. Disease-modifying therapies continue to face development challenges due to the blood–brain barrier (BBB), which prevents more than 98% of small molecules and all biologics from entering the central nervous system. The therapeutic landscape for neurodegenerative diseases has recently undergone transformation through advances in targeted drug delivery that include ligand-decorated nanoparticles, bispecific antibody shuttles, focused ultrasound-mediated BBB modulation, intranasal exosomes, and mRNA lipid nanoparticles. This review provides an analysis of the molecular pathways that cause major neurodegenerative diseases, discusses the physiological and physicochemical barriers to drug delivery to the brain, and reviews the most recent drug targeting strategies including receptor-mediated transcytosis, cell-based “Trojan horse” approaches, gene-editing vectors, and spatiotemporally controlled physical methods. The review also critically evaluates the limitations such as immunogenicity, scalability, and clinical translation challenges, proposing potential solutions to enhance therapeutic efficacy. The recent clinical trials are assessed in detail, and current and future trends are discussed, including artificial intelligence (AI)-based carrier engineering, combination therapy, and precision neuro-nanomedicine. The successful translation of these innovations into effective treatments for patients with neurodegenerative diseases will require essential interdisciplinary collaboration between neuroscientists, pharmaceutics experts, clinicians, and regulators.

## 1. Introduction

**Figure 1:**
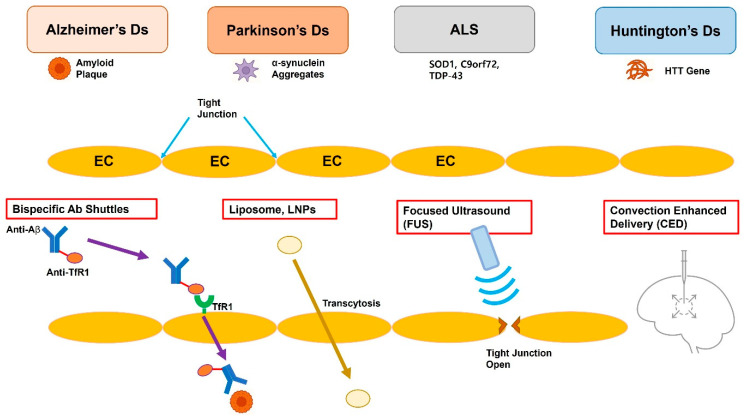
Schematic representation of the blood–brain barrier (BBB) and four clinically relevant strategies for targeted drug delivery to the central nervous system. The top row lists four prevalent neurodegenerative disorders—Alzheimer’s disease (AD), Parkinson’s disease (PD), amyotrophic lateral sclerosis (ALS), and Huntington’s disease (HD)—all of which require therapeutic access to the central nervous system. (1) Bispecific antibody shuttles engage endothelial receptors such as transferrin or IGF-1R and undergo receptor-mediated transcytosis, ferrying otherwise impermeable macromolecular cargo into the brain parenchyma. (2) Liposomes or lipid nanoparticles (LNPs) traverse the BBB via adsorptive or ligand-directed endocytosis/transcytosis. (3) Focused ultrasound (FUS), in conjunction with circulating microbubbles, induces transient and reversible opening of endothelial tight junctions, enabling size-independent passive diffusion of therapeutics. (4) Convection-Enhanced Delivery (CED) utilizes a stereotactically placed micro-catheter to infuse agents directly into the interstitial space, driving bulk flow that distributes the drug over centimeter-scale volumes independent of molecular weight. EC, endothelial cell; TfR, transferrin receptor; Aβ, β-amyloid; SOD1, superoxide dismutase 1; C9orf72, chromosome 9 open reading frame 72; TDP-43, TAR DNA-binding protein 43; HTT, huntingtin.

Neurodegeneration comprises a group of progressive, incurable diseases that lead to selective neuronal death, protein misfolding, neuroinflammation, and circuit dysfunction [1]. Population aging increases the number of people affected, with the World Health Organization (WHO) predicting that dementia cases will triple by 2050 and Parkinson’s disease (PD) increasing faster than any other serious neurological condition [2,3]. The current pharmacotherapy, including levodopa, acetylcholinesterase inhibitors, and riluzole, primarily treats the symptoms and has decreasing efficacy with time [4]. The fundamental challenge common among all neurodegenerative therapeutics lies not merely in target identification, but in achieving sufficient brain exposure to engage their target effectively. The main obstacle to developing drugs for the central nervous system (CNS) is the blood–brain barrier (BBB), which is a highly specialized cerebrovascular endothelium that contains tight junctions, efflux transporters, and metabolic enzymes that exclude most xenobiotics [5,6]. The medicinal chemistry approach is useful, but most of the time, it cannot produce molecules that have all the required characteristics of potency, safety, manufacturability, and barrier penetration [7]. This limitation has catalyzed a paradigm shift toward engineering-enabled delivery strategies that represent a convergence of nanotechnology, molecular biology, and precision medicine principles. Thus, pharmaceutical science has moved towards engineering-enabled delivery strategies that either (a) bypass intact barriers by using endogenous transport pathways (e.g., transferrin receptor and insulin receptor) or (b) create temporary breaches in the barrier under image guidance [8,9]. Advances in RNA therapeutics and gene editing and programmable protein degraders have produced a number of macromolecular payloads whose clinical efficacy depends on their ability to cross the BBB [10]. Rather than presenting these approaches as isolated technologies, this review integrates them within a unifying framework of precision neurotherapeutics, where delivery strategy selection depends on disease pathophysiology, target accessibility, and patient-specific factors. This review gives a comprehensive but balanced review of the targeted therapies and delivery platforms that are expected to revolutionize neurodegenerative disease treatment strategies (Figure 1). Preclinical mechanistic discoveries are integrated with translational biomaterials science, and pitfalls from recent clinical trials are discussed together with recommendations for future CNS therapeutics [11].

## 2. Pathophysiology and Therapeutic Targets

Understanding the molecular pathogenesis of neurodegenerative diseases is critical for rational delivery system design, as target accessibility, tissue distribution, and therapeutic windows vary dramatically across different pathological contexts.

### 2.1. Alzheimer’s Disease (AD)

AD pathogenesis involves β-amyloid (Aβ) accumulation, tau hyper-phosphorylation, synaptic loss, glial activation, and metabolic insufficiency [12]. Traditional targets like β- and γ-secretase have yielded limited clinical success, prompting the exploration of novel pathways. Apart from the traditional β- and γ-secretase inhibition, new targets are emerging, which include soluble Aβ oligomers, tau kinases (glycogen synthase kinase-3β (GSK-3β) and cyclin-dependent kinase 5 (CDK5)), microglial Triggering Receptor Expressed on Myeloid cells 2 (TREM2), and the NOD-, LRR-, and pyrin domain-containing protein 3 (NLRP3) inflammasome [13,14,15]. The spatial distribution of these targets—from extracellular plaques to intraneuronal tau tangles to microglial inflammasomes—demands delivery systems capable of multi-compartmental targeting. The plaque-clearing monoclonal antibodies lecanemab and donanemab show only modest cognitive effects, which points to the need for earlier intervention and improved drug delivery to the CNS [16,17].

### 2.2. Parkinson’s Disease (PD)

PD is characterized by substantia nigra dopaminergic neuron degeneration along with α-synuclein (encoded by the SNCA gene) aggregation, mitochondrial impairment (involving PTEN-induced kinase 1 (PINK1) and PARK7, also known as DJ-1), lysosomal dysfunction (involving Glucocerebrosidase 1 (GBA1)), and neuroinflammation [18,19]. Emerging therapies aim to modify disease progression beyond symptomatic relief.

New disease-modifying concepts include small-molecule glucocerebrosidase chaperones, antisense oligonucleotides (ASOs) targeting SNCA mRNA, ketogenic metabolic modulators, and gene-therapy vectors delivering neurotrophic factors (glial cell line-derived neurotrophic factor (GDNF) and neurturin) [20,21,22]. The anatomical specificity of PD pathology in discrete brainstem nuclei creates opportunities for targeted delivery approaches that concentrate therapeutics in the affected regions while minimizing systemic exposure.

### 2.3. Amyotrophic Lateral Sclerosis (ALS)

ALS causes the death of upper and lower motor neurons through several converging mechanisms including excitotoxic glutamate, RNA foci (due to chromosome 9 open reading frame 72 (C9orf72)), misfolded superoxide dismutase 1 (SOD1), and TAR DNA-binding protein 43 (TDP-43) pathology [23]. The drugs riluzole and edaravone provide minimal survival benefit [24]. The precision approach includes ASOs (e.g., tofersen) for mutant SOD1 silencing and CRISPR-Cas13d RNA editing of C9orf72 repeat expansions [25,26,27]. The success of tofersen demonstrates that targeted nucleic acid delivery to motor neurons is achievable, providing a proof-of-concept for RNA-based precision medicine in neurodegeneration.

### 2.4. Huntington’s Disease (HD)

The huntingtin (HTT) gene contains CAG trinucleotide expansions that generate poly-glutamine aggregates, which can cause neurodegeneration in striatal areas [28]. ASOs such as WVE-003, along with dual-adeno-associated virus (AAV) CRISPR approaches, targets mutant HTT expression while preserving normal copies of the gene [29,30]. The genetic basis of HD offers unique opportunities for allele-specific targeting, where delivery systems must discriminate between mutant and wild-type huntingtin with high fidelity.

### 2.5. Other Neurodegenerative Diseases

Multiple-system atrophy and frontotemporal dementia, along with prion diseases, share similar proteostatic and neuroimmune alterations that create additional opportunities for targeted approaches to aggregate protein clearance, autophagy regulation, and immunometabolic reprogramming [31] (Table 1). These disorders collectively demonstrate that while disease-specific pathways vary, common themes of protein aggregation, neuroinflammation, and cellular stress responses provide convergent targets for platform delivery technologies.

The diversity of therapeutic targets across neurodegenerative diseases necessitates flexible delivery platforms capable of accommodating different payload types—from small molecules targeting kinases to large biologics clearing protein aggregates to nucleic acids modulating gene expression. This molecular complexity underscores why no single delivery approach can address all CNS therapeutic needs, but rather demands a precision medicine framework where the delivery strategy selection matches the target characteristics and disease pathophysiology.

## 3. Barriers to CNS Drug Delivery

The BBB represents more than a simple anatomical obstacle; it is a dynamic, multi-layered system whose barrier properties vary with disease state, anatomical location, and temporal factors. Understanding these variations is crucial for developing targeted delivery strategies that exploit barrier vulnerabilities while respecting physiological constraints.

### 3.1. Blood–Brain Barrier (BBB)

The BBB is formed by brain endothelial cell tight junctions (claudin-5 and occludin), which unite with pericytes and astrocytic end-feet and create a specialized basement membrane [6]. This complex structure limits paracellular transport, requiring targeted delivery approaches for CNS drugs. The BBB maintains a low paracellular permeability (<10^−8^ cm s^−1^ for sucrose) while displaying a high density of efflux transporters including P-glycoprotein (P-gp) and breast cancer resistance protein (BCRP) to block xenobiotic entry [5,6]. However, this barrier is not uniformly impermeable; specific transport systems evolved to meet brain metabolic demands and provide potential entry points for therapeutics. The three receptors transferrin receptor (TfR), insulin receptor (IR), and low-density lipoprotein receptor-related protein 1 (LRP1) facilitate restricted macromolecular transport through receptor-mediated transcytosis [32]. Critically, these receptor densities vary across brain regions and are dysregulated in neurodegeneration, creating disease-specific opportunities and challenges for receptor-targeted delivery.

### 3.2. Blood–CSF Barrier and Glymphatic Pathways

The choroid plexus epithelium contains leaky fenestrated capillaries but has tight epithelial junctions that create a secondary barrier to the cerebrospinal fluid (CSF) [33]. This anatomical distinction is therapeutically relevant because drugs delivered via CSF can access periventricular brain regions through bulk flow mechanisms that bypass the BBB entirely. Therapeutic opportunities exist through the manipulation of meningeal lymphatic and glymphatic networks that conduct macromolecular clearance [34]. The recent recognition of glymphatic dysfunction in neurodegeneration suggests that delivery strategies must account for altered clearance patterns that could affect drug residence times and distributions [35].

### 3.3. Disease-Specific Barrier Modulation

An emerging paradigm in CNS drug delivery recognizes that neurodegenerative diseases themselves alter barrier properties in ways that can be exploited therapeutically. In AD, regional BBB breakdown occurs in areas of high amyloid deposition, creating “leaky” zones that may allow for enhanced drug penetration [36]. Similarly, neuroinflammation in multiple sclerosis and PD increases BBB permeability through inflammatory mediator release [37,38]. Understanding these disease-induced barrier modifications enables the development of pathology-targeted delivery strategies that leverage disease-specific vulnerabilities rather than fighting against intact barriers.

### 3.4. Physicochemical Constraints

Therapeutic brain exposure for small molecules requires a molar mass of less than 400 Da, a calculated logP (clogP) score between 2 and 4, a polar surface area (PSA) smaller than 70 Å^2^, and a neutral charge [7]. Macromolecules consisting of proteins and RNAs surpass these limits so they require delivery through carrier-mediated systems or invasive delivery methods [39]. These constraints, known as Lipinski’s “Rule of Five” adaptations for CNS drugs, explain why traditional medicinal chemistry approaches have limited success with complex neurotherapeutics like protein aggregation inhibitors or gene therapies [40].

The integration of barrier properties with physicochemical constraints creates a three-dimensional optimization challenge where delivery system design must simultaneously address molecular size, charge, stability, and targeting specificity. This complexity has driven the evolution of simple drug modification approaches toward sophisticated multi-component delivery platforms that can navigate multiple barrier layers while maintaining payload integrity.

## 4. Targeted Drug-Delivery Strategies

The field of CNS drug delivery has evolved from a focus on individual technologies toward integrated platform approaches that combine multiple mechanisms to achieve therapeutic brain exposure. This section examines these strategies not as isolated solutions, but as components of a precision delivery toolkit where the selection of a strategy depends on the target characteristics, the disease pathophysiology, and patient factors.

### 4.1. Medicinal Chemistry Optimization

BBB-penetrant therapeutics have reached their highest level of development through medicinal chemistry strategies [41]. The novel paradigms in CNS multiparameter optimization (MPO) scoring have become more popular than traditional methods [42]. While these approaches remain foundational, their limitations with macromolecular therapeutics have necessitated the platform approaches discussed subsequently. Soft drug design features metabolically sensitive elements which transform potent CNS-targeting drugs into inactive compounds after systemic exposure, thus enhancing their therapeutic benefits [43]. Researchers have developed dopamine-β-hydroxylase inhibitors that contain para-nitrophenyl carbonate masks, which break down quickly in plasma to prevent peripheral sympatholysis [44]. Targeted prodrugs leverage nutrient and peptide transporters as their delivery mechanism [45]. The addition of L-tyrosine–valine esterification has enabled the transport of kynurenine-3-mono-oxygenase inhibitors, which are impermeable on their own, through the BBB [46]. The brain uptake of reporter positron emission tomography (PET) tracers increased by 8 times compared to the parent drug, but this effect disappeared when L-type amino acid transporter 1 (LAT1) was knocked down [46]. The field now favors structural changes in drugs that avoid P-gp substrate patterns instead of using pharmacological pump blockers because these may cause systemic toxicity and drug interactions [41]. However, medicinal chemistry approaches face fundamental limitations when applied to the new generation of neurotherapeutics. Protein therapeutics, nucleic acids, and complex small molecule combinations cannot be optimized using traditional structure–activity relationships, creating an imperative for the development of delivery platform technologies.

### 4.2. Nanoparticle Platforms

Nanoparticle-based delivery represents the most rapidly advancing area of CNS therapeutics, driven by the convergence of materials science, targeting biology, and manufacturing innovations. Rather than viewing different nanoparticle types as competing technologies, the field is moving toward rational platform selection based on payload requirements, target accessibility, and safety constraints.

#### 4.2.1. Lipid Nanoparticles (LNPs)

The LNP renaissance propelled by mRNA vaccines has catalyzed CNS applications [10]. The design of LNPs, including liposomes, begins with their lipid composition, which determines their stability and cellular uptake. The use of ionizable lipids which exhibit an acid dissociation constant (pKa) of ~6.2 enables reduced protein adsorption at body pH while allowing for endosomal escape through acidification [39]. To enhance BBB penetration, LNPs are functionalized with targeting ligands, bridging lipid chemistry to specific receptor interactions. Brain-targeted LNPs incorporate targeting elements such as apolipoprotein E-mimetic peptides, anti-TfR antibody fragments, or peptides like angiopep-2 to enhance brain uptake compared to non-targeted LNPs [47]. This targeted approach has shown promise in preclinical models, paving the way for clinical translation. Several mRNA-LNP candidates for replacing deficient proteins in neurological disorders are now advancing to clinical trials and have shown promising preclinical CSF/blood concentration ratios [48]. However, the clinical success of LNPs is tempered by challenges such as cationic lipid toxicity, which can induce inflammatory responses and suboptimal targeting efficiency in vivo, with only a fraction of nanoparticles reaching the CNS [39].

**Liposomal formulations**, a well-established subset of LNPs, have high biocompatibility and the ability to encapsulate both hydrophilic and hydrophobic drugs [49]. Early studies suggested that conventional liposomes are largely excluded from a healthy BBB; however, low-level penetration (~0.05% ID·g^−1^) can be therapeutically meaningful for highly potent drugs [50]. PEGylated liposomes decorated with anti-TfR antibodies have shown enhanced delivery of anti-Aβ antibodies in Alzheimer’s disease models, achieving a 3-fold increase in brain uptake compared to non-targeted liposomes [51]. Stealth liposomes have also been repurposed for curcumin delivery, leading to a 35% reduction in the amyloid plaque burden after four weekly iv injections [52]. However, liposomes face challenges such as limited endosomal escape compared to ionizable LNPs, requiring further optimization of their lipid composition [53].

The evolution of LNP technology demonstrates how rational design can overcome initial limitations through iterative improvement. However, the field must address fundamental questions about long-term safety and targeting specificity before these platforms can achieve their full therapeutic potential.

#### 4.2.2. Polymeric Nanoparticles

The clinical implementation of polymeric carriers depends on achieving Good Manufacturing Practice (GMP) scalability and maintaining a consistent size distribution [54]. Building on material design, these nanoparticles are engineered with targeting ligands to enhance BBB crossing. Poly(lactic-co-glycolic acid)–polyethylene glycol (PLGA-PEG) nanospheres that delivered rapamycin and displayed rabies virus glycoprotein 29 (RVG29) on their surface successfully reduced cortical microgliosis by 60% and restored novel-object-recognition abilities in 5xFAD mice, a model for AD [55]. This success highlights their potential, but their material properties also introduce challenges. Scientists have developed PLGA cores with zwitterionic poly(carboxybetaine) brushes to prevent complement activation and thereby eliminated the first-dose infusion problems that appeared in previous clinical trials [56]. However, polymeric nanoparticles often suffer from lower targeting efficiency compared to LNPs and face challenges in achieving a uniform size, which complicates their large-scale production [54]. The trade-offs between polymer biodegradability, drug loading, and targeting efficiency illustrate the complex optimization required for successful clinical translation.

#### 4.2.3. Inorganic and Hybrid Carriers

The surface area of mesoporous silica nanoparticles (MSNs) exceeds 700 m^2^g^−1^, which enables the simultaneous delivery of oligonucleotides and small-molecule kinase inhibitors with different hydrophilic and hydrophobic properties [57]. This versatility is useful in applications requiring precise control, such as photothermal therapy. The combination of gold nanorod-silica yolk–shell hybrids enables researchers to monitor photothermal effects in real time during 808 nm near-infrared laser stimulation of brain-derived neurotrophic factor (BDNF) plasmid release, which promotes neurite outgrowth in human induced pluripotent stem cell (iPSC)-derived cortical neurons [58]. External control mechanisms further extend their utility. The external control of deep-brain structures becomes possible through the use of magnetic iron(II,III) oxide (Fe_3_O_4_) cores [59]. However, inorganic carriers face significant translational barriers, including potential long-term tissue accumulation and complex synthesis processes that hinder their scalability [57]. While inorganic carriers offer unique capabilities for controlled release and multi-modal imaging, their clinical translation remains limited by biocompatibility concerns and their manufacturing complexity.

#### 4.2.4. Exosomes and Biomimetic Vesicles

The natural “don’t eat me” signals (e.g., CD47) from mesenchymal stem cell (MSC)-derived exosomes enhance their circulation duration beyond 6 h [60]. Exosomes carrying catalase delivered through intranasal administration reduced nigrostriatal reactive oxygen species (ROS) levels and maintained tyrosine hydroxylase levels in rotenone-induced PD rats [61]. Advancing from natural exosomes, synthetic designs can improve payload capacity. The development of “designer exosomes” combines synthetic lipidoid membranes with native tetraspanin scaffolds to achieve a high payload capacity and minimal immunogenicity [62]. Their targeted delivery potential has been demonstrated in stroke models. The addition of a cyclic arginylglycylaspartic acid-tyrosine-lysine (RGDyK) peptide to vesicles doubled the amount of particles found at lesion sites during transient middle cerebral artery occlusion (tMCAO) stroke experiments compared to unmodified vesicles [63]. However, their low production yields and batch variability limit their scalability, posing challenges for clinical translation [60]. The promise of exosome-based delivery lies in their natural biocompatibility, but realizing this potential requires innovations in scalable production and standardization.

#### 4.2.5. Nanogels and Hydrogel-Derived Carriers

Nanogels assembled from cholesterol-modified pullulan and phenylalanine-functionalized poly(ethylene glycol) could hold a >90% water content while maintaining a <120 nm hydrodynamic diameter [54]. The porous network of these systems can effectively encapsulate Aβ-targeting ASOs with >95% efficiency [64]. The incorporation of matrix metalloproteinase-9 (MMP-9) crosslinkers into the system allows for targeted site-specific release since this enzyme becomes up-regulated in BBB-disrupted peri-infarct zones [65]. Their clinical potential had been demonstrated in preclinical models. The administration of Aβ_42_-targeting ASOs via intravenous delivery during weekly sessions decreased soluble Aβ_42_ levels by 38% while restoring the synaptic density within the dentate gyrus of AD mice [65]. However, achieving uniform drug release and preventing in vivo degradation remain critical challenges [64].

#### 4.2.6. Stimuli-Responsive Composite Systems

ROS-cleavable thioketal linkers, pH-labile β-thioester bonds, and enzyme-sensitive peptides allow for on-demand release [66]. These mechanisms are particularly effective in neurodegenerative environments with altered biochemistry. The nanovectors can penetrate tauopathy extracellular matrices due to their porous silicon microparticle structure which releases ultrasmall (<10 nm) ceria–zirconia cores [67]. The combination of ROS scavenging with tau-kinase inhibition produced better Y-maze alternation scores in rTg4510 mice through synergistic action [67]. Despite their promise, these systems face challenges in achieving consistent release in complex brain environments.

#### 4.2.7. Theranostic and Multimodal Nanocarriers

Theranostic carriers integrate diagnostic and therapeutic (Rx) capabilities [68]. Liposomes containing gadolinium chelates transport manganese sulfate for T1-weighted magnetic resonance imaging (MRI), which allow for real-time monitoring of target engagement [69]. This dual functionality supports clinical translation by providing actionable data. LNP photoacoustic probes can serve as depth-sensitive tools to monitor mRNA translation throughout the cortical layers of the brain [70] (Table 2). However, balancing the diagnostic and therapeutic capabilities introduces regulatory and design complexities [68].

The nanoparticle platform landscape demonstrates remarkable diversity in addressing different aspects of CNS delivery challenges. However, this diversity also highlights the need for rational selection criteria based on specific therapeutic requirements rather than pursuing platform technologies in isolation.

### 4.3. Ligand-Directed and Bispecific Antibody Shuttles

Bispecific antibodies represent a convergence of immunotherapy and delivery science, offering exquisite target specificity combined with active transport mechanisms. Their clinical advancement provides important insights into the challenges of and opportunities for receptor-mediated transcytosis approaches. The development of molecular engineering methods now enables the modification of affinity strength as well as valency and epitope placement to favor transcytosis over endosomal degradation [71]. For instance, optimizing the TfR-binding affinity enhances brain penetration but risks immune activation. The mean brain concentration of the bispecific antibody RG6102 reached 1.1 μg g^−1^, which was five times lower than that of gantenerumab [72]. Mutations within the neonatal Fc receptor (FcRn)-binding domains of albumin (“Abdeg”) increase the natural albumin pathway while maintaining safety and doubling the area-under-the-curve (AUC) [73]. This advance can be applied to broader applications, such as siRNA delivery. Prion protein (PrP)-targeting small interfering RNA (siRNA) constructs decorated with aptamers can achieve picomolar binding affinity without triggering innate immune responses [74], and angiopep-2 peptide-drug conjugates have entered phase I trials for conditions such as neuropathic pain [75]. The success of bispecific antibodies in achieving measurable brain exposure validates receptor-mediated transcytosis as a viable clinical strategy while also revealing the complex optimization required for balancing transcytosis efficiency and safety.

### 4.4. Gene and RNA Therapies

The emergence of nucleic acid therapeutics has created both new opportunities and unique delivery challenges for CNS applications. Unlike small molecules, these macromolecular therapeutics require delivery systems that can protect the payload integrity while facilitating cellular uptake and subcellular trafficking.

#### 4.4.1. Recombinant AAV Vectors

The screening of bar-coded capsid libraries using humanized BBB models revealed that AAV-LK03 and AAV-MYO5 exhibit a 6- to 10-fold higher neuron preference than AAV9 [76]. These advances enable precise CNS targeting, but immune responses remain a challenge. The first participants received AB-1005 (AAV2-GDNF) injections via MRI-guided Convection-Enhanced Delivery (CED) in the AskBio phase II trials in late 2024 (REGENERATE-PD) [77]. An AAV2-GDNF clinical trial (NCT06285643) tested parallel dose-escalation of putaminal infusion volumes that were optimized through real-time convection MRI [78].

#### 4.4.2. Non-Viral and Synthetic Vectors

Lipid–polyethyleneimine hybrid nanoplexes together with charge-reversal poly(β-amino ester) polyplexes can evade innate capsid immunity, although they have historically displayed limited expression duration [79]. The incorporation of Venezuelan equine encephalitis (VEE) replicons, which can replicate themselves, resulted in weeks-long protein synthesis [80].

#### 4.4.3. Self-Amplifying RNA and Circular RNA

Vaccine-originated self-amplifying RNA (saRNA) LNPs can allow for a 10- to 100-fold reduction in the required dosage for administration [81]. The intravenous delivery of an saRNA that encodes a soluble N-terminal tau decoy resulted in a 45% decrease in insoluble tau in P301S mice while minimizing increases in hepatic transaminase levels [82]. Circular RNA (circRNA) functions as an innate sensor evader and can maintain a half-life of more than 48 h, which enabled durable grip strength recovery in preclinical ALS models after receiving circ-micro-dystrophin treatment [83].

#### 4.4.4. Genome and Epigenome Editing

The combination of *Streptococcus pyogenes* Cas9-High Fidelity 1 (SpCas9-HF1) with adenine base editor 8e (ABE8e) delivered through split-intein dual-AAV configurations enables the correction of PD-related GBA1 variant mutations in patient-derived neurons by restoring lysosomal β-glucocerebrosidase activity [84]. The epigenetic editing system consisting of a deactivated Cas9 fused to a Krüppel-associated box and methyl-CpG-binding protein 2 (dCas9-KRAB-MeCP2) provides a safe method for silencing mutant huntingtin while avoiding DNA breakage which helps prevent trinucleotide repeat instability [85].

The rapid clinical advancement of nucleic acid therapeutics demonstrates their transformative potential while simultaneously revealing the critical importance of delivery system optimization for achieving therapeutic efficacy with acceptable safety profiles.

### 4.5. Cell-Mediated “Trojan Horse” Delivery

Cell-based delivery represents the most biologically sophisticated approach to CNS therapeutics, leveraging living systems’ natural ability to cross biological barriers and respond to local microenvironmental cues. However, this sophistication introduces unique challenges in manufacturing, characterization, and regulatory oversight. Chimeric Antigen Receptor (CAR) macrophages that express high levels of Interleukin-10 (IL-10) migrate to α-synuclein plaque locations where they locally suppress tumor necrosis factor-alpha (TNF-α) and Interleukin-1 beta (IL-1β) signaling gradients [86]. The combination of closed-system bioreactors with Cluster of Differentiation 14 (CD14) magnetic separation enables the manufacturing of clinical-grade products that can achieve >90% purity after 72 h [87]. Neural stem cell (NSC) carriers that produce neprilysin protease can break down Aβ outside of cells, leading to a 20% survival increase in APP/PS1dE9 mice [88]. The main regulatory issues consist of biodistribution tracking via zirconium-89 immuno-PET and methods to prevent insertional mutagenesis through thymidine kinase/ganciclovir (TK/GCV) suicide-gene protections [89]. While cell-mediated delivery offers unparalleled biological sophistication, its clinical translation requires addressing fundamental questions about cell fate, safety monitoring, and manufacturing scalability that differ substantially from traditional drug development paradigms.

### 4.6. Physical and Regional Techniques

Physical delivery methods represent a complementary approach that can enhance the efficacy of other delivery strategies by temporarily overcoming barrier constraints or providing direct access to target tissues. These techniques are particularly valuable for delivering therapeutics that cannot be adequately modified for passive BBB penetration.

#### 4.6.1. Focused Ultrasound (FUS) + Microbubbles

Modern FUS arrays utilize phased-array beam steering alongside real-time cavitation mapping systems that operate at frequencies between 230 kHz and 1.5 MHz [90]. The six-cycle bilateral frontal BBB-opening phase II trial showed 95% technical success along with no parenchymal hemorrhage in 66 mild AD patients while the exploratory cognitive results indicated slower disease progression [91]. The combination of FUS with lecanemab infusions demonstrated that the sonicated areas had a 38% reduction in plaques compared to control hemispheres [92]. The FUS technique produces astrocyte and microglial phenotypic changes that lead to A2/M2 neuroprotective states that could enhance combinatorial immunomodulatory effects [93]. The two main FUS techniques for brain exposure use (i) single-element helmet-based systems that cover entire hemispheres with reduced precision at the millimeter scale and (ii) intraconal stereotactic arrays which create precise sub-millimeter foci in deep-brain nuclei including the thalamus and putamen [90]. The formulation choice (lipid-based vs. perfluorocarbon shell microbubbles) determines both the threshold for cavitation and the level of safety during procedures [90]. Non-human primates showed no astrogliosis or cognitive deficits when the BBB was opened once per week for 8 weeks [94]. The clinical success of FUS demonstrates the viability of physical BBB modulation approaches while also highlighting the importance of precise spatiotemporal control in minimizing off-target effects.

#### 4.6.2. Convection-Enhanced Delivery (CED)

Novel reflux-resistant stepped cannulae allow for flow rates up to 10 μL min^−1^ without backflow [95]. Real-time MR-theranostic tracking of a gadolinium–albumin surrogate demonstrated homogeneous infusate dissemination across 80% of the putamen [96]. AAV2-aromatic L-amino acid decarboxylase (AADC) received through CED resulted in stable 3-fold ^18^F-fluoro-L-dopa PET signals while maintaining Unified Parkinson’s Disease Rating Scale (UPDRS) improvements during a 60-month observation period [97]. Research on frontotemporal dementia and ALS patients uses intra-cortical CED methods because spinal CSF mixing remains limited when using intrathecal ASOs [98].

#### 4.6.3. Electromagnetic, Photothermal, and Magnetothermal Modalities

Alternating magnetic field (AMF) stimulation of magnetite–silica composites heats transient receptor potential vanilloid 1 (TRPV1)-expressing dorsal striatal neurons, achieving reversible motor recovery in PD mice without macroscale tissue heating [99]. The process of photochemical internalization (PCI) utilizes porphyrin derivatives along with low-intensity light to break endo-lysosomal membranes which then release the enclosed macromolecules [100] (Table 3).

Physical delivery methods demonstrate the potential for precise spatiotemporal control of drug delivery, but their clinical adoption requires careful consideration of their invasiveness, repeatability, and long-term safety implications.

### 4.7. Integrative Analysis of Delivery Approaches

Rather than viewing these delivery strategies as competing technologies, the field is evolving toward precision medicine approaches where method selection depends on the disease characteristics, target properties, and patient factors. This requires a systematic comparison of the key performance metrics of the delivery platforms. To guide therapeutic development, a comparative analysis of the delivery systems is essential (Table 4). Lipid nanoparticles, including liposomes, excel in encapsulating nucleic acids (e.g., mRNA and siRNA) with high efficiency (up to 95%) and are scalable for clinical use, but their cationic lipid components may cause toxicity, and their brain penetration remains moderate (CSF/blood ratios of 0.1–0.5) [39,48]. This moderate penetration, while seemingly low, may be therapeutically sufficient for highly potent payloads, illustrating the importance of considering delivery efficiency in the context of the payload potency. Exosomes offer biocompatibility and natural targeting via CD47 signals, but their production is limited by low yields (10^10^ particles/mL) and variability [60]. The production limitations of exosomes highlight a recurring theme in advanced delivery technologies: the trade-off between biological sophistication and manufacturing scalability. Polymeric nanoparticles provide versatile surface modification but face challenges in GMP scalability and complement activation [54]. Bispecific antibodies can achieve high specificity via receptor-mediated transcytosis (e.g., TfR and IR) but are costly and may trigger immune responses [72]. FUS enables transient BBB opening with high precision (sub-millimeter foci) but carries the risk of microhemorrhages and requires expensive equipment [90].

The diversity of performance characteristics of the different delivery platforms suggests that combination approaches may be necessary to achieve optimal therapeutic outcomes. For instance, FUS-mediated BBB opening could enhance the brain penetration of targeted nanoparticles, while bispecific antibodies could improve the specificity of physical delivery methods.

### 4.8. Emerging Combination Strategies and Platform Integration

The field’s evolution toward combination approaches reflects a growing recognition that neurodegenerative diseases’ complexity demands multi-modal therapeutic interventions. Sequential delivery strategies, for example, FUS-mediated BBB opening followed by targeted nanoparticle administration, have shown synergistic effects in preclinical models, achieving brain drug concentrations 10-fold higher than the individual approaches alone [101,102,103,104]. Similarly, the integration of diagnostic imaging with therapeutic delivery—such as MRI-guided FUS combined with real-time pharmacokinetic monitoring—enables precise dosing and treatment optimization [105,106]. These combination strategies also address the temporal dynamics of neurodegeneration, where different pathological processes may require distinct delivery approaches at different disease stages [107].

This comparative analysis reveals that no single delivery approach can address all CNS therapeutic needs. Instead, the field must develop decision frameworks that match delivery strategy selection to specific therapeutic requirements, disease characteristics, and patient factors. This precision medicine approach to delivery system selection represents the next evolution in CNS drug development.

## 5. Clinical Translation and Regulatory Landscape

The translation of advanced CNS delivery systems from preclinical success to clinical reality requires navigating complex regulatory, manufacturing, and commercial challenges that differ substantially from traditional small molecule development. Understanding these challenges is critical for realistic assessments of delivery technology potential.

### 5.1. Approved or Conditionally Approved Agents

The U.S. Food and Drug Administration (FDA) has granted regulatory approval to five CNS biologics, namely aducanumab, lecanemab, donanemab, tofersen, and nusinersen, since 2021 [16,17,25]. Despite intense public debate about surrogate endpoints, the FDA’s accelerated-approval process continues to value biomarker shifts, including amyloid PET and CSF neurofilament light chain levels, when the unmet need is severe [108]. These approvals establish important precedents for the types of evidence required for CNS delivery system approval, particularly regarding the acceptance of biomarker endpoints and the role of delivery enhancement in achieving therapeutic efficacy. Pharmaceutical companies must monitor amyloid-related imaging abnormalities (ARIA) and develop complex pharmacokinetic–pharmacodynamic (PK–PD) models for sustained market access after receiving post-market commitments [109].

### 5.2. Late-Stage Pipeline

The Roche product RG6102 and Eli Lilly’s anti-TfR/tau format entered multicenter phase II clinical trials as bispecific antibody shuttles in early 2025, with their first interim results scheduled for 2027 [72]. AskBio’s AB-1005 (AAV2-GDNF via CED) and Voyager/Sanofi’s AAV-microRNA targeting SNCA are two leading gene therapy candidates [77,110]. The latter drug avoids innate immune activation through a microglia-detargeting capsid design [110]. Research studies used MRI to guide medication delivery into plaque-enriched cortical areas while BBB opening was used in combination with lecanemab, donanemab, or ASO RGX-104 [92]. These combination approaches represent a critical evolution in clinical trial design, where delivery enhancement is integrated with therapeutic evaluation rather than being developed in isolation.

### 5.3. Safety, Immunogenicity, and CMC Considerations

The complexity of advanced delivery systems introduces novel safety and manufacturing challenges that require new regulatory frameworks and risk assessment approaches.

#### 5.3.1. Immunogenicity

The prevalence of anti-AAV neutralizing antibodies persists in 35–60% of adult patients, which necessitates that the patients either meet specific criteria or undergo plasmapheresis procedures [111]. PEG-specific IgM can trigger complement activation-related pseudo-allergy (CARPA) during LNP infusions, which can be prevented through the use of PEG alternatives like poly(2-hydroxyethyl aspartamide) (pHEA) and graded desensitization methods [112]. These immunogenicity challenges illustrate the need for patient stratification strategies and alternative formulation approaches during clinical development.

#### 5.3.2. Genotoxicity

The delivery of CRISPR editors through LNPs raises safety concerns regarding chromosomal translocations which has led to the development of GUIDE-seq and whole-genome duplex sequencing methods for nanogram-scale DNA analysis [113]. The development of sophisticated safety assessment tools for gene editing delivery demonstrates the regulatory system’s adaptability to novel therapeutic modalities.

#### 5.3.3. Chemistry, Controls, and Manufacturing (CCM)

Regulatory bodies now require digital batch records, together with real-time release testing [114]. The FDA tightened the acceptable polydispersity index (PDI) for genome-editing therapeutics to <0.20 in their latest draft guidance (January 2024) [114]. These evolving CCM requirements reflect the growing regulatory sophistication in handling complex delivery systems while also highlighting the additional development costs and timelines associated with meeting these standards.

### 5.4. Scaling Up Manufacturing and Supply Chains 

LNP production has shifted from ethanol-injection microfluidics to acoustic mixing reactors, which can produce >200 g RNA per batch for phase III trial purposes [115]. The use of producer cell lines with inducible RepCap expression enables AAV vector yields to reach 2 × 10^17^ vector genomes (vg) from a 2000 L bioreactor without needing transient transfection processes [116]. The expense of maintaining cold-chain logistics is USD 50 million per product launch; thus, major cost reductions are possible through the use of 4 °C stable lyophilized LNP “cakes” that last >12 months [117].

These manufacturing innovations demonstrate the field’s maturation from laboratory-scale production to commercial manufacturing, but also highlight the substantial infrastructure investments required for successful clinical and commercial deployment.

### 5.5. Health Economics and Reimbursement

Cost-effectiveness analyses (CEAs) determine lecanemab has an incremental cost-effectiveness ratio (ICER) of ~USD 200,000 per quality-adjusted life-year (QALY) under the present pricing, which exceeds the standard willingness-to-pay thresholds [118]. The U.S. healthcare system conducts tests of value-based contracts that base reimbursement on amyloid PET results and delayed nursing-home admissions [119]. The prices for single CED gene therapy administrations often exceed USD 1 million, but national health systems are developing payment models based on annuity and milestone approaches [120].

The health economics of advanced delivery systems present fundamental challenges to healthcare accessibility, requiring innovative reimbursement models that balance innovation incentives with patient access. These economic considerations may ultimately determine which delivery technologies achieve widespread clinical adoption.

### 5.6. Evolution of Regulatory Frameworks 

The regulatory landscape for CNS delivery systems is rapidly evolving to address the unique challenges posed by these complex therapeutic modalities. The European Medicines Agency (EMA) released its Advanced Therapy Medicinal Product (ATMP) guideline revision in June 2024 to fully align genome-editing drug potency assays with the FDA’s genome-editing draft guidance by requiring at least 70% target specificity and complete off-target profiling reports [114,121]. The new pilot program enables simultaneous scientific advice meetings between different regulatory bodies, which could speed up global clinical trial start-ups. This regulatory harmonization reflects the global nature of CNS delivery system development and the need for coordinated oversight approaches.

However, significant challenges remain in adapting regulatory frameworks designed for traditional pharmaceuticals to complex delivery systems. Issues such as combination product regulation, personalized medicine approaches, and long-term safety monitoring require continued regulatory innovation.

### 5.7. Digital Health Integration and Real-World Evidence

The integration of digital health technologies with CNS delivery systems represents an emerging frontier that could transform clinical development and post-market surveillance. Wearable devices and smartphone apps can now monitor sleep patterns, cognitive function, and motor symptoms in real time, providing continuous assessments of therapeutic efficacy [122]. Digital biomarkers derived from speech patterns, gait analysis, and cognitive tasks offer objective, sensitive measures of neurological function that complement traditional clinical scales [123,124]. This real-world evidence generation is particularly valuable for CNS therapeutics, where patient-reported outcomes and functional assessments are critical endpoints. The FDA’s recent guidance on digital therapeutics and real-world evidence provides a framework for incorporating these technologies into regulatory decision-making, potentially accelerating the approval and optimization of complex delivery systems [125,126].

The clinical translation landscape demonstrates both the promise and challenges of advanced CNS delivery systems. While regulatory frameworks are adapting to accommodate these technologies, successful translation requires careful attention to safety, manufacturing, and economic considerations that may ultimately determine which promising preclinical approaches achieve a clinical impact.

## 6. Future Perspectives and Integrative Approaches

The future of CNS drug delivery lies not in the dominance of any single technology, but in the intelligent integration of multiple approaches to create precision therapeutic strategies. This integration represents a fundamental shift from technology-centric to patient-centric development paradigms. The convergence of artificial intelligence (AI), advanced materials science, and precision medicine is creating unprecedented opportunities for CNS therapeutic development.

### 6.1. AI-Guided Carrier Design

The use of generative models which combine physicochemical features with receptor expression atlases and in silico fluid dynamics will improve ligand selection and formulation optimization [70,127]. This computational approach promises to accelerate the traditionally empirical process of delivery system optimization by predicting optimal formulations before experimental testing.

### 6.2. Multiplexed, “All-in-One” Nanomedicine

A single LNP can combine anti-Aβ siRNA with a tau kinase inhibitor alongside neurotrophic mRNA to treat multiple aspects of AD pathogenesis [64]. Orthogonal release triggers (pH and ROS) enable spatiotemporal drug sequencing [66]. This multiplexed approach addresses the multifactorial nature of neurodegeneration more comprehensively than single-target therapies, potentially improving efficacy while reducing treatment complexity.

### 6.3. Personalized Exosome Therapy

Autologous iPSC-derived neuron exosomes loaded with patient-specific microRNAs may help reduce immune rejection and improve therapeutic precision [128]. The convergence of personalized medicine with delivery technology offers the potential for truly individualized neurotherapeutics, though it also introduces substantial manufacturing and regulatory complexity.

### 6.4. Regenerative Genome Editing

Base-editing and prime-editing tools that correct pathogenic alleles directly in cells may achieve one-time cures, provided that delivery methods are developed for efficiency and scar-free application [67]. The promise of curative single-dose therapies represents the ultimate goal of precision medicine, but they require delivery systems capable of achieving near-perfect efficiency and safety.

### 6.5. Neuro-Immune Modulation

Nanoparticle delivery of mammalian target of rapamycin (mTOR) or NLRP3 reprogramming agents to microglia for phenotypic conversion could potentially work better with protein-clearance methods [86,129]. Innovation in neurodegenerative diseases is likely to be driven by the convergence of immunology and pharmaceutics over the next decade, as our understanding of neuroinflammation’s role in disease progression deepens.

The integration of these emerging approaches suggests a future where CNS therapeutics are designed as comprehensive, personalized treatment platforms rather than single-target interventions. This evolution requires new frameworks for clinical development, regulatory oversight, and healthcare delivery that can accommodate the complexity and potential of integrated precision neurotherapeutics.

## 7. Conclusions

The landscape of CNS drug delivery has undergone a fundamental transformation, from viewing the blood–brain barrier as an insurmountable obstacle to recognizing it as a sophisticated biological system whose properties can be leveraged for therapeutic advantage. The development of targeted drug delivery systems, which include rational small-molecule design, smart nanocarriers, bispecific antibody shuttles, gene-editing vectors, and image-guided BBB modulation methods, has begun to break down the past obstacles in treating neurodegenerative diseases. The BBB is now thought of as a biological rather than an insurmountable barrier, as proven by the successful laboratory and clinical tests with tofersen (SOD1-ALS), LNP-mRNA progranulin restoration, and focused-ultrasound-facilitated antibody therapy [25,48,92]. However, the true promise of these technologies lies not in their individual capabilities, but in their potential for intelligent integration within precision medicine frameworks. Human neurodegenerative diseases require adaptable treatment platforms that ensure both safety and scalability. The field’s evolution from single-modality approaches toward combination strategies reflects a deeper understanding of neurodegeneration’s complexity and the need for multi-target therapeutic interventions.

The critical path forward requires addressing several key challenges: (i) the development of rational selection criteria for matching delivery approaches to specific therapeutic requirements, disease characteristics, and patient factors; (ii) the creation of manufacturing and regulatory frameworks capable of handling the complexity of integrated delivery systems while maintaining safety and accessibility; and (iii) the establishment of health economic models that balance innovation incentives with patient access considerations.

Perhaps most importantly, the field must embrace a systems-level perspective that recognizes the interdependence of delivery technologies, disease biology, and clinical applications. The successful implementation of laboratory breakthroughs into routine clinical practice requires rigorous pharmacokinetic–pharmacodynamic modeling, transparent benefit–risk assessments, as well as collaborative regulatory science. This systems approach extends beyond technical considerations to encompass the full ecosystem of stakeholders—patients, clinicians, regulators, and payers—whose collective needs must be addressed for successful translation. The convergence of advanced delivery technologies with precision medicine principles offers unprecedented opportunities to transform the treatment of neurodegenerative diseases. Advanced pharmaceutics that incorporates precision medicine principles will enable the development of targeted therapies to transform the lives of millions affected by neurodegenerative diseases.

## Figures and Tables

**Table 1:** Key molecular targets and representative investigational therapeutics for major neurodegenerative diseases.

Disease	Therapeutic Target(s)	Representative Agent/Strategy	Key Limitation(s)	Ref.
**AD**	• Extracellular Aβ Plaques	**Lecanemab, donanemab** (anti-Aβ monoclonal antibodies)	• Modest cognitive benefit	[16,17]
			• Risk of ARIA	
	• Intracellular Tau Tangles	**ASOs targeting Tau** (e.g., BIIB080/IONIS-MAPTRx)	• Requires intrathecal delivery	[13]
			• Long-term efficacy unknown	
**PD**	• α-Synuclein Aggregates	**ASOs** (antisense oligonucleotides) **targeting SNCA**	• Unable to deliver to deep brain nuclei	[21]
	• GBA1 Enzyme Deficiency		• Systemic side effects	
	• GDNF Replacement	**AAV2-GDNF** (gene therapy via CED)	• Invasive delivery (CED)	[22]
			• Pre-existing AAV immunity	
**ALS**	• Mutant SOD1 Protein	**Tofersen** (SOD1-targeting ASO)	• Benefits limited to specific genetic subtype	[25]
			• Modest survival extension	
	• C9orf72 Repeat Expansions	**CRISPR-Cas13d** (RNA editing)	• Off-target editing risks	[27]
			• In vivo delivery challenges	
**HD**	• Mutant Huntingtin (mHTT)	**WVE-003** (allele-selective ASO)	• Difficulty balancing mHTT knockdown and normal HTT preservation	[29]
	• HTT Gene	**CRISPR/Cas9** (dual-AAV gene editing)	• Risk of permanent off-target DNA changes	[30]
			• AAV delivery limitations	

AD: Alzheimer’s disease; PD: Parkinson’s disease; ALS: amyotrophic lateral sclerosis; HD: Huntington’s disease; Aβ: β-amyloid; ARIA: amyloid-related imaging abnormalities; ASO: antisense oligonucleotide; SNCA: synuclein alpha gene; GBA1: Glucocerebrosidase 1; GDNF: glial cell line-derived neurotrophic factor; AAV: adeno-associated virus; CED: Convection-Enhanced Delivery; SOD1: superoxide dismutase 1; HTT: huntingtin.

**Table 2:** Overview of nanoparticle-based platforms for CNS drug delivery.

Nanoparticle Platform	Key Features and Payloads	Advantages	Key Challenges	Refs.
**Lipid Nanoparticles (LNPs)**	• Ionizable lipids, PEGylation • Payloads: mRNA, siRNA, ASOs	• High encapsulation efficiency • Clinically validated and scalable	• Potential lipid toxicity • Moderate brain penetration	[39,47,48]
**Polymeric Nanoparticles**	• PLGA, PLA cores; surface ligands • Payloads: Small molecules, proteins	• Tunable drug release • High biocompatibility	• Lower payload capacity compared to LNPs • Potential for complement activation	[54,56]
**Exosomes/Biomimetic**	• Natural cell-derived vesicles • Payloads: Endogenous cargo, loaded drugs	• Low immunogenicity • Natural BBB-crossing ability	• Low production yield and purity • Batch-to-batch variability	[60,62]
**Inorganic/Hybrid Carriers**	• Gold, silica, iron oxide cores • Payloads: Drugs, imaging agents	• Theranostic capabilities • Externally triggerable release	• Long-term tissue accumulation • Complex, non-scalable synthesis	[57,59]

CNS: central nervous system; LNP: lipid nanoparticle; PEG: polyethylene glycol; mRNA: messenger RNA; siRNA: small interfering RNA; ASO: antisense oligonucleotide; PLGA: poly(lactic-co-glycolic acid); PLA: polylactic acid; BBB: blood–brain barrier.

**Table 3:** Physical and regional techniques for enhancing CNS drug delivery.

Technique	Mechanism of Action	Key Advantages	Key Challenges	Ref(s).
**Focused Ultrasound (FUS)**	• Microbubble-assisted transient opening of tight junctions	• Non-invasive and targeted • Reversible and repeatable	• Risk of microhemorrhage • High equipment cost	[90,91,92]
**Convection-Enhanced Delivery (CED)**	• Direct, pressure-driven infusion into brain parenchyma	• Bypasses the BBB entirely • High local drug concentration	• Highly invasive (surgical) • Limited to focal areas	[95,97]
**Intranasal Delivery**	• Bypasses BBB via olfactory and trigeminal neural pathways	• Non-invasive, patient-friendly • Rapid onset of action	• Low and variable bioavailability (10–30%) • Limited to specific molecules	[61]
**Magnetothermal/Photothermal**	• Nanoparticle-mediated local heating to increase permeability or trigger release	• High spatiotemporal control • On-demand drug release	• Requires co-delivered nanoparticles • Risk of tissue heating/damage	[99,100]

CNS: central nervous system; BBB: blood–brain barrier.

**Table 4:** Comparison of major CNS delivery platforms.

Delivery Platform	Primary BBB-Crossing Mechanism	Typical Brain Penetration	Main Strength	Primary Weakness	Refs.
**Lipid/Polymeric Nanoparticles**	Receptor-Mediated Transcytosis	Low to moderate	**Versatility:** Can carry diverse payloads (RNA, small molecules)	**Efficiency:** Low percentage of injected dose reaches brain	[47,54]
**Bispecific Antibodies**	Receptor-Mediated Transcytosis	Moderate to high	**Specificity:** High-affinity targeting of both BBB receptors and brain target	**Complexity:** High production cost and potential immunogenicity	[71,72]
**AAV Gene Therapy**	Transduction of Endothelial Cells/Neurons	High (with direct injection)	**Durability:** Potential for long-term or one-time treatment	**Safety:** Pre-existing immunity and genotoxicity risks	[76]
**Focused Ultrasound (FUS)**	Physical Disruption of Tight Junctions	High (in targeted region)	**Universality:** Enables delivery of nearly any systemic agent	**Safety/Cost:** Risk of hemorrhage and high equipment cost	[90,92]
**Cell-Mediated Delivery**	Active Migration Across BBB	Variable	**Bio-integration:** “Living drugs” can respond to microenvironment	**Control:** Difficult to control cell fate, distribution, and safety	[86,88]

CNS: central nervous system; BBB: blood–brain barrier; AAV: adeno-associated virus.

## Abbreviations

WHO	World Health Organization
PD	Parkinson’s disease
CNS	central nervous system
BBB	blood–brain barrier
AD	Alzheimer’s disease
Aβ	β-amyloid
GSK-3β	glycogen synthase kinase-3β
CDK5	cyclin-dependent kinase 5
TREM2	Triggering Receptor Expressed on Myeloid cells 2
NLRP3	NOD-, LRR-, and pyrin domain-containing protein 3
SNCA	synuclein alpha gene
PINK1	PTEN-induced kinase 1
GBA1	Glucocerebrosidase 1
ASOs	antisense oligonucleotides
GDNF	glial cell line-derived neurotrophic factor
ALS	amyotrophic lateral sclerosis
C9orf72	chromosome 9 open reading frame 72
SOD1	superoxide dismutase 1
TDP-43	TAR DNA-binding protein 43
HD	Huntington’s disease
HTT	huntingtin
AAV	adeno-associated virus
P-gp	P-glycoprotein
BCRP	breast cancer resistance protein
TfR	transferrin receptor
IR	insulin receptor
LRP1	low-density lipoprotein receptor-related protein 1
CSF	cerebrospinal fluid
clogP	calculated logP
PSA	polar surface area
MPO	multiparameter optimization
PET	positron emission tomography
LAT1	L-type amino acid transporter 1
LNPs	lipid nanoparticles
pKa	acid dissociation constant
GMP	Good Manufacturing Practices
PLGA-PEG	poly(lactic-co-glycolic acid)–polyethylene glycol
RVG29	rabies virus glycoprotein 29
MSNs	mesoporous silica nanoparticles
BDNF	brain-derived neurotrophic factor
iPSC	induced pluripotent stem cell
Fe_3_O_4_	iron(II,III) oxide
MSC	mesenchymal stem cell
ROS	reactive oxygen species
RGDyK	arginylglycylaspartic acid-tyrosine-lysine
tMCAO	transient middle cerebral artery occlusion
MMP-9	matrix metalloproteinase-9
Rx	diagnostic and therapeutic
MRI	magnetic resonance imaging
FcRn	neonatal Fc receptor
AUC	area-under-the-curve
PrP	prion protein
siRNA	small interfering RNA
CED	Convection-Enhanced Delivery
VEE	Venezuelan equine encephalitis
saRNA	self-amplifying RNA
circRNA	circular RNA
SpCas9-HF1	*Streptococcus pyogenes* Cas9-High Fidelity 1
ABE8e	adenine base editor 8e
dCas9-KRAB-MeCP2	deactivated Cas9 fused to a Krüppel-associated box and methyl-CpG-binding protein 2
CAR	Chimeric Antigen Receptor
IL-10	Interleukin-10
TNF-α	tumor necrosis factor-alpha
IL-1β	Interleukin-1 beta
CD14	Cluster of Differentiation 14
NSC	neural stem cell
TK/GCV	thymidine kinase/ganciclovir
FUS	focused ultrasound
AADC	aromatic L-amino acid decarboxylase
UPDRS	Unified Parkinson’s Disease Rating Scale
AMF	alternating magnetic field
TRPV1	transient receptor potential vanilloid 1
PCI	photochemical internalization
FDA	U.S. Food and Drug Administration
ARIA	amyloid-related imaging abnormalities
PK–PD	pharmacokinetic–pharmacodynamic
CARPA	complement activation-related pseudo-allergy
pHEA	poly(2-hydroxyethyl aspartamide)
CCM	Chemistry, Controls, and Manufacturing
PDI	polydispersity index
vg	vector genomes
CEAs	cost-effectiveness analyses
ICER	incremental cost-effectiveness ratio
QALY	quality-adjusted life-year
EMA	European Medicines Agency
ATMP	Advanced Therapy Medicinal Product
AI	artificial intelligence
